# How do women experience a change in their clinically-derived breast cancer risk estimates: views from a UK family history risk and prevention clinic

**DOI:** 10.1186/s12885-026-15651-w

**Published:** 2026-01-29

**Authors:** Victoria G. Woof, Anthony Howell, Lynne Fox, Lorna McWilliams, D. Gareth Evans, David P. French

**Affiliations:** 1 University of Manchester, 1.13 Coupland 1 Oxford Road, Manchester, M13 9PL UK; 2 Family History Risk and Prevention Clinic, Prevent Breast Cancer Unit, Manchester University NHS Foundation Trust, Manchester, UK

**Keywords:** Breast cancer, Breast cancer risk, Cancer prevention, Early detection, Updated risk estimates, Qualitative

## Abstract

**Background:**

Introducing breast density and polygenic risk scores into breast cancer prediction models results in greater precision and can involve alterations to previously communicated risk estimates and preventative management. This study explored how women from a UK family history risk and prevention clinic view, experience and understand a change in their communicated risk.

**Methods:**

Twenty-two women were interviewed; 11 received an increased risk and 11 a decreased risk. Data were analysed using reflexive thematic analysis.

**Results:**

Four themes were generated: *(i) unaware of possibility of risk change*, illustrating women believed their risk estimates would remain unaltered due to their family history, hence receiving a lower risk was shocking but a relief, but an increased risk somewhat unsurprising, *(ii) a trusted source influences adapted risk appraisals*, highlighting the clinic’s reputation as an information source, as well as personal connections with the service effecting risk appraisals, *(iii) perceived value of new risk factor knowledge*, where women contemplated the usefulness of knowing their breast density and polygenic risk scores, *(iv) heart versus head: changes in preventative management*, where the implications of an updated risk estimate was processed.

**Conclusions:**

Women reacted positively to their updated breast cancer risk estimates and trusted the information provided, even when preventative management options changed.

**Supplementary Information:**

The online version contains supplementary material available at 10.1186/s12885-026-15651-w.

## Background

In the UK, personalised estimation of breast cancer risk is only provided to those with a strong family history of the disease (e.g. those with affected first or second-degree relatives who meet guidelines for referral [1]) outside of research settings. Such women may be referred by primary or secondary care for genetic testing and/or to family history risk and prevention clinics (FHRPCs) to determine their lifetime and 10-year risk of developing breast cancer.

To calculate breast cancer risk, multifactorial risk prediction models such as the Tyrer-Cuzick [2, 3] and CanRisk [4] are used. These models include risk factors such as, family history, body mass index, hormonal and reproductive factors (e.g., age at menarche, hormone replacement therapy (HRT) or oral contraceptive use, age at first full term pregnancy and age of menopause). Diet and health behaviours (i.e. alcohol intake) are also incorporated. Recently the validity and discriminatory capabilities of these risk prediction models have improved significantly due to the inclusion of new risk factors, breast density (ratio of fibro-glandular tissue to fat in the breast) and a polygenic risk score (PRS; a calculation of genetic variants (single nucleotide polymorphisms (SNPs)) related to hereditary breast cancer) [5–10]. Consequently, it is likely that these risk factors will be used in clinical settings in the future, as their inclusion would provide more precise risk estimates and treatment stratification [11].

Presently, women at an above-average (moderate; 5–7.99.99%) or high (> 8%) 10-year risk of developing breast cancer are offered additional screening up to age 60 years, health behaviour change advice, and preventive medication in line with National Institute of Health and Clinical Excellence (NICE) clinical guidelines [1]. If updated risk estimates are communicated to those attending FHRPCs, a major challenge will be the inclusion of new risk factors causing changes to previously communicated risk estimates. This could mean that for some, eligibility for early detection and preventative management changes. For example, those who experience a risk reduction may not be eligible for continued annual screening.

A recent systematic review of qualitative research [12] found that clinically-derived breast cancer risk estimates were often not in line with personal risk appraisals. Reasons for this included incongruence between clinical estimates and women’s personal expectations and women’s varied understanding and knowledge about breast cancer risk factors [12]. Similarly, a study with women from the general population who received notification of an increased breast cancer risk demonstrated that the nature of personal experiences of breast cancer in others, difficulties attributing breast cancer causes, personal expectations of risk and the perceived usefulness of knowing one’s risk all contributed to how women personally appraised their breast cancer risk, in spite of *objective* clinical notification [13].

To date, there appears to be a paucity of research investigating how changes to a personal estimate of disease risk is viewed, understood or experienced for any disease group. As breast cancer risk prediction becomes more precise, it will be essential to understand how women who experience a change in their risk estimates perceive this change and whether new risk information is incorporated into pre-existing breast cancer risk appraisals. This is particularly important if women experience a change in their risk management. The present research addresses this issue by examining views from women taking part in the Family History Risk (FH-Risk) study [14, 15] who had the opportunity to receive an updated risk based on all known (to date) risk factors in 2022-23. The aim of the present study therefore was to assess how those who received an update and were notified of a change in their breast cancer risk, view, experience and understand this change.

## Methods

### Design

This study employed a cross-sectional qualitative design employing semi-structured one-to-one telephone and Zoom interviews.

### Participants and setting

Women for the present study were sampled from the original FH-Risk study [14, 15] which recruited in 2010-12. Women who participated had already received their risk of developing breast cancer as part of routine practice at the FHRPC between 1990 and 2012 and were under follow-up. The purpose of the original FH-Risk study was to validate breast density and a PRS into risk prediction models, like the Tyrer-Cuzick model. Women who took part consented to have their mammogram images used and provided a blood sample for the purpose of calculating a PRS. Providing risk feedback to women as part of this study was not the original aim, however some women did receive information on the potential effects of SNP18 (a PRS based on a set of 18 SNPs associated with breast cancer. These 18 SNPs were chosen at this time as they accounted for around two-thirds of the familial risk component [14]) on their previously counselled breast cancer risk estimates. This was only an indication that it may have increased if their PRS was above 2.0 or reduced if below 0.5. These cutoffs were chosen at this time as they were defensible clinically, given the state of knowledge regarding how SNP18 contributed to overall breast cancer risk. However, the information women received was partially incomplete with the great majority receiving no indication of a change in risk [14, 15]. Additionally, although providing consent for the use of their mammogram images, women were not given breast density feedback, as prior to 2013 breast density was not included in risk prediction models. Moderate and high penetrance genes were also not explored in the original study.

As a PRS and breast density have now been validated and incorporated into popular risk prediction models, the original FH-Risk research team deemed it appropriate to give women who participated in this study the opportunity to learn of their updated risk estimates. Therefore, women who participated in FH-Risk and who were either discharged based on NICE clinical guidelines [1] or remained under follow-up were given the opportunity (via postal invite) in 2022/23 to be informed of their updated breast cancer risk. If interested, a FHRPC consultant arranged a telephone consultation to discuss this update, with a minority of consultations provided in person when COVID-19 restrictions were lifted. The same consultant provided all risk feedback, with summary letters distributed after each consultation. Summary letters included details of what was discussed and an image to demonstrate women’s breast density. If preventative medication was offered, women also received the appropriate leaflets. To discuss the complexity of SNPs/a PRS the consultant used an analogy in consultations to aid understanding (see supplementary file for summary letter and analogy example).

Women who opted to receive their updated breast cancer risk were provided feedback based on the mean risk of the Tyrer-Cuzick [2, 3] and CanRisk models [4], incorporating a PRS (based on a panel of 313 SNPs) [9, 16] and breast density (measured using BIRADS™) [17]. As part of the models, women also received pathogenic variant feedback on 12 high and moderate penetrance genes linked to breast cancer, including *BRCA1/2* [18]. Incorporating a PRS and breast density into risk prediction models resulted in women’s breast cancer risk increasing, decreasing or staying the same. This meant that for some entitlement for additional screening changed, as well as eligibility for preventative medication.

To be eligible for the present study, women needed to have been notified of a change (increase or decrease in risk and/or change in risk category [1]/early detection and preventative management) in their breast cancer risk and test negative on 12 high and moderate penetrance genes. A negative test for these genes was a requirement as the experiences of receiving positive test results was considered potentially different to those who were non-carriers, due to experiencing different clinical pathways, as well as the potential for women to reflect on the implications for family members when considering their own genetic testing decisions. Furthermore, as this study aimed to assess the experience of risk changing due to the addition of breast density and a PRS, a PRS is extremely unlikely to change a risk score in women who test positive for a high or moderate risk gene. Therefore, it was decided that women with these high and moderate risk genes were excluded from this study. Women with a personal breast cancer history were also excluded.

### Interview procedure

Women who received their updated risk feedback were sent a study invite and participant information sheet 1 to 2 months after receiving consultation summary letters. Reminder invite letters were sent 2 to 3 weeks after initial invites. One-to-one telephone or Zoom interviews were arranged with those willing to discuss their updated risk and consultation experience. Prior to interviewing, women provided audio-recorded verbal informed consent. Women were also asked demographic questions (see Table 1). A semi-structured flexible topic guide was used throughout the interviews (see supplementary file). A Patient and Public Involvement and Engagement (PPIE) group comprised of seven women with experience of receiving clinically-derived breast cancer risk estimates from the Predicting Risk of Cancer at Screening (PROCAS) study [15] helped develop questions and topic areas for the topic guide, including the order questions were asked in and the phrasing used. This was to ensure that questions related meaningfully to participants and the experience of receiving breast cancer risk feedback. Questions focused on the following topics, (i) women’s breast cancer risk appraisals prior to updated risk feedback, (ii) women’s thoughts on their updated risk consultation, (iii) women’s understandings of their risk changing (these questions aimed to further the literature on where risk appraisals following the communication of a clinically-derived risk estimate are adaptable to change and congruent with this clinical estimate) and (iv) women’s views on their risk management (see supplementary file). Interviews lasted 28–90 min (M = 51.1, SD = 17.6), were recorded, transcribed verbatim by an external agency and pseudonymised. Recruitment ceased when the research team believed there was sufficient data to answer the research question [20].

### Ethical considerations

Given the potentially sensitive nature of discussing breast cancer risk, a distress protocol was in place. This outlined how the research team would respond if participants became upset during the interview. Two women did become distressed, not in relation to their risk estimate but while reflecting on experiences of breast cancer within their families. In both cases, interviews were paused, participants were offered the option to stop completely or reschedule, and both chose to continue.

For the minority of women who expressed uncertainty about aspects of their consultation or preventative management, the interviewer (VGW) encouraged them to contact their clinical team directly. This process was built into the ethics application, and women had established relationships with their clinicians, which facilitated follow-up when needed. Throughout the research process, the team was reflexive about balancing the need to generate insights with the ethical responsibility to minimise unnecessary worry or burden for participants.

### Analysis

Data were analysed in NVivo12 using reflexive thematic analysis [21] approached from a critical realist perspective. This perspective assumes that a reality exists independently of human perceptions or beliefs, but that our access to it is always partial, mediated through subjective sense-making, social contexts, and normative frameworks [22]. Analysis began with the primary author (VGW; who also conducted the interviews) re-familiarising herself with women’s experiences by reading and re-reading transcripts. First readings took place alongside listening to the corresponding audio recording. On subsequent re-reads notes were taken of initial ideas and patterns in the transcripts. Following this, initial coding was approached inductively, meaning that codes produced were reflective of the ideas and patterns in the dataset. These initial codes were descriptive, where the primary author (VGW) aimed to capture the nuance in data. Both semantic and latent level coding was used. The researchers chose to combine these coding approaches as it ensured that both the explicit and implicit meanings within the data were captured, with semantic coding capturing women’s direct expressions and meanings and latent coding enabling the researchers to go beyond this surface level detail to explore the underlying themes, assumptions and contexts that shape women’s experiences. Coding was iterative, with codes refined, re-labelled and collapsed as new data were analysed. Codes were then analysed together and combined to form patterns, which represented meaningful experiences and ideas in the dataset that related meaningfully to the research question. Initial candidate themes were discussed within the research team and refined until the final thematic structure was established.

## Results

### Interview participants

Twenty-two women were interviewed, 10 over Zoom and 12 via telephone. Table 1 shows sample demographics and study data. Table 2 indicates changes in 10-year breast cancer risk for each woman.

**Table 1:** Participant demographic and study data

Demographics and study data	*N*
Age (Mean)	47–68 years (56.7 years)
Ethnicity	
*White British*	21
*Jewish American*	1
Index of Multiple Deprivation decile (Mean)	1–10 (6)*
Education	
*Postgraduate qualification*,* i.e. Masters*,* PhD*	7
*Degree*	5
*‘A’ levels/other post-16 qualifications at college*	5
*‘O’ Levels/GCSEs*	4
*No qualifications*	1

* Higher number meaning living in least deprived areas of the UK [19]

**Table 2:** Change in 10-year breast cancer risk and management recommendations following the inclusion of a PRS and breast density

Participant	TC/CR (10Y) Mean*	TC/CR + PRS & BD (10Y) Mean**	Previous risk management recommendations	Updated risk management recommendations
Decreased risk (D)				
Kerry	8.4	4.55	Annual screening	3-yearly screening
Violet	12.5	7	Prescribed preventative medication; annual screening	Continue preventative medication; continue annual screening
Melissa	5.65	3.15	3-yearly screening	Continue 3-yearly screening
Alison	6.45	2.7	Annual screening	3-yearly screening
Becky	11.5	5.75	3-yearly screening	Continue 3-yearly screening
Grace	5.5	2.9	Annual screening	3-yearly screening
Natalie	7.7	2.3	3-yearly screening	Continue 3-yearly screening
Tracy	10.75	3.8	Annual screening	3-yearly screening
Stef	19.6	6.45	Annual screening	Continue annual screening
Rose	6.3	2.4	Annual screening	3-yearly screening
Leanne	11.4	4.8	Prescribed preventative medication; annual screening	Cease preventative medication; 3-yearly screening
Increased risk (I)				
Jenny	5.75	8.5	Annual screening	Continue annual screening
Alexa	9.8	17.5	Annual screening	Consider preventative medication; continue annual screening
Hayley	9	12.7	Annual screening	Consider preventative medication; continue annual screening
Paula	7.45	21.75	3-yearly screening	Consider preventative medication; annual screening
Pamela	9.45	14.2	3-yearly screening	Consider preventative medication; annual screening
Harriet	11.05	16.85	Annual screening	Consider preventative medication; continue annual screening
Lisa	10.75	11.6	3-yearly screening	Consider preventative medication; annual screening
Abigail	7.35	10.4	Annual screening	Consider preventative medication; continue annual screening
Bronwen	6.8	9.75	Prescribed preventative medication; annual screening	Continue preventive medication; continue annual screening
Frances	5.35	6.6	Annual screening	Consider preventative medication; continue annual screening
Hannah	6.25	14.8	3-yearly screening	Consider preventative medication; annual screening

*Mean 10-year (10Y) risk of the Tyrer-Cuzick (TC) and CanRisk (CR) models, excluding a PRS and breast density (BD)

**Mean 10-year (10Y) risk of the Tyrer-Cuzick (TC) and CanRisk (CR models, including a PRS and breast density (BD)

### Reflexive thematic analysis

Four themes were generated, *(i) unaware of possibility of risk change*,* (ii) a trusted source influences adapted risk appraisals*,* (iii) perceived value of new risk factor knowledge and (iv) heart versus head: changes in preventative management.* After each pseudonymised quote (meaning all participants have been assigned new names) a ‘D’ or an ‘I’ is used to indicate whether a decrease or an increase in risk was experienced.

### Unaware of possibility of risk change

Only a minority of women had an awareness prior to receiving their updated breast cancer risk that certain risk factors (i.e. health behaviour/diet changes) could change their estimated risk. Nevertheless, the majority did not seem to possess strong ideas as to whether risk could change, describing never consciously thinking about their risk changing:

*No*,* actually I probably didn’t think it would change*,* no*,* it didn’t really cross my mind that one*,* actually… Abigail (I)*.

Most women believed their risk would remain the same throughout their lifetime due to their family history, as they were not aware of any factors that would affect their risk as significantly. When approached by the clinic to receive a more precise risk estimate, it was only at this point where questions about whether risk could change were considered, with women describing feeling curious. Many acknowledged being motivated to seek a risk update due to the impact it could have on their children’s risk, whilst others expressed an interest in hearing about advances in the field. Although having no pre-conceived expectations as to whether their risk had changed, all women described wanting to receive an update in spite of the outcome or implications:

*…of course I want to know. I want to know whether it’s good or I want to know whether it’s bad. I need to know. So*,* there was no hesitation in my mind… Lisa (I)*.

Notification of a risk reduction was described as shocking for some. For these women, shock appeared related to the strength of their family history, surprised that their risk could be reduced so dramatically with the inclusion of new risk factors. For Grace, receiving a negative genetic test challenged her expectations and beliefs about her risk, which she had held all her life:

*…I was expecting to be carrying the gene*,* and I was expecting to be told that I’m at high-risk*,* so I was quite shocked when I was told that I wasn’t*,* and I was low-risk*,* I wasn’t expecting that at all. Obviously*,* I was happy about it*,* but I wasn’t expecting it…Grace (D)*

Although initially finding a reduced risk a shock, women revealed feeling relieved, with Stef describing it as *‘a weight lifted’*. Conversely, those informed of an increase to their risk were disappointed, but not overly troubled by this news. This is potentially attributable to these women having always ‘lived’ at increased risk of breast cancer, so although risk had increased, this knowledge did not appear to worry them significantly, with Hayley (I) identifying that her risk was *‘one in four and I’ve gone to one in three*,* so it’s not a massively different risk’.*

Jenny surmised that her risk could potentially change again, indicating her acceptance of the fluidity of risk estimation and her understanding that a clinically-derived breast cancer risk is not definitive:

*In two years’ time they might come across something and say oh*,* we were wrong*,* your risk factor is virtually zero […] I think because I know that there could be change*,* I’m aware that my risk factors could change. Jenny (I)*.

This view was also discussed by others, who argued for regular updates if risk were to change frequently, especially if taking preventative medication or if new risk factors are discovered. Overall, however, women appeared to have little prior concern about their risk consultations and the potential for risk changing.

### A trusted source influences adapted risk appraisal

From their initial visit to the FHRPC all women recalled feeling protected by the service and grateful for annual screening. The clinic seemed to provide women with a sense of belonging and community, with consistency of staff, personalised care and a positive atmosphere being highly valued. Attending clinic annually became ‘*routine*’ for some and looked forward to their annual engagement:

*…I really felt cared for and looked after and part of a family [at the FHRPC]*,* because as I say*,* I’ve always trusted [name of clinician]*,* the people I’ve met there*,* you know? I’ve always felt that they had my best interests at heart. Becky (D)*

With this in mind, when receiving their updated breast cancer risk estimates women appeared positive and trusted the new estimate provided. Women’s trust in the service and the value attributed to it also seemed to influence the ease in which new risk estimates were internalised and accepted. Integration of new risk factors, breast density and a PRS seemed to be accomplished with relative ease, accepting the clinician’s knowledge and re-defining personal risk appraisals in line with this information:

*…the things that really stuck with me that we talked about were…that I didn’t have any of the genetic markers [high-penetrance genes]*,* but they looked at 300 markers [SNPs] on my DNA*,* and that’s where I had the higher risk*,* that’s where the higher risk came from. Harriet (I)*

The majority of women described their risk as changed and attributed changes to breast density and a PRS, as well as results informing them of their non-carrier status for pathogenic variants in 12 high-penetrance breast cancer genes. Following communication of this information there appeared to be a shift in the majority of women’s understandings of their risk from their initial FHRPC visit, where a family history and the assumption of being a gene mutation carrier were considered the key drivers in their risk. Following their updated risk notification, women described appreciating the impact of these new risk factors, whilst also processing past assumptions about their genetic risk:

*I had probably assumed and probably wrongly before I knew that there wasn’t a genetic link. What I hadn’t appreciated was some of the other factors still added up to the higher than average risk […] So again*,* let’s say I was surprised. I wasn’t particularly upset*,* but I was maybe surprised. But that’s always because I’d worked on an assumption…Bronwen (I)*

Following notification of their updated risk, women openly discussed understanding that low or high breast density, or, a low or high PRS resulted in risk reducing or increasing. Women described understanding the ‘take home message’ of this information and felt they had sufficient knowledge to enable them to integrate this information into an improved understanding of risk:

*…he explained to me about how my breast density had changed since I started on the programme years ago and they’d become much more denser and now I was in the most dense category*,* and that obviously increased my risk…Alexa (I)*

Developing a ‘take home message’ or ‘gist’ understanding of breast density and PRS information seemed to be associated with the way these risk factors were communicated. Specifically, an analogy used to explain a PRS/SNPs, as well as a visual representation of breast density was described as helpful in enabling women to understand the contribution of these risk factors to their updated risk:

*And then we talked about the SNPs bit*,* and he told me about how that works for your gene and the A’s and the Bs and I have more B’s than A’s which was also very good news. That was useful. Alison (D)*

These techniques and positive relationships with the clinic seemed to contribute to *most* personal risk appraisals being in line with the updated estimate provided.

### Perceived value of new risk factor knowledge

All women mentioned breast density, a PRS and their non-carrier status for pathogenic variants when describing their change in risk. Non-carrier status for pathogenic variants in high-penetrance genes seemed to be particularly significant, with women elated by their result, not only for themselves but for their children. For some this information and a PRS was considered simultaneously. By doing so, attention attributed to their PRS appeared reduced, due to the overwhelming affective response associated with their non-carrier status. Instead, women appeared to possess a vague but mostly accurate understanding of their PRS and its contribution to their risk, and seemed satisfied with this level of knowledge. When considering the usefulness of knowing their PRS, some explained the futility of dwelling on the score, due to their inability to control it:

*I can only manage the factors that I can manage […] The fact that they’re [SNPs] there*,* I can’t change them*,* so I’m not going to overly worry about it. Bronwen (I)*

Breast density results were considered in more depth. When reflecting on their knowledge of breast density prior to attending their risk consultations, women explained holding vague understandings, with most explanations containing inaccuracies and misunderstandings:

*What I thought was*,* like there was less liquid in there*,* not fat*,* but more…what’s the word I’m looking for? More just human tissue rather than them being big and bloated with air and fat and water*,* you know*,* liquid? Becky (D)*

Accuracy of knowledge still remained mixed after receiving an updated risk estimate. Women instead focused on the overall contribution of their breast density to their risk and as with knowledge for SNPs/a PRS, appeared satisfied with ‘gist’ knowledge:

*As small as they are [her breasts]*,* you know*,* they are made of matter that is not great in terms of breast cancer. You know*,* that’s how I read it*,* that’s how I feel about it…Lisa (I)*

Most women acknowledged the usefulness of knowing their breast density, with some considering or pursuing preventative medication to reduce it. Communication of breast density was also considered important more widely, with women advocating that all eligible woman should be given the opportunity to know their breast density. However, women identified some caveats to breast density communication. Specifically, they questioned the usefulness of breast density information if nothing conclusive can be done to manage it. Additionally, the usefulness of providing breast density information in isolation of other known risk factors was also considered.

### Heart versus head: changes in preventative management

For some women still under follow-up at the FHRPC, their updated risk estimates resulted in them being ineligible for annual screening. This was due to their risk reducing below the level recommended for additional screening in NICE guidelines. This appeared to cause conflict emotionally, with women understanding they were no longer eligible and accepting this, yet feeling a sense of loss from being discharged. Despite being pleased that risk had reduced, losing the protectiveness and comfort the service offers was challenging to reconcile:

*…when he said I’d go to the three [yearly screening]*,* that’s just the bit that made me think a bit. And I’ve not got to think like that but it’s just made me think*,* oh*,* crikey*,* yeah*,* a bit*,* makes me a bit more nervous now of it [breast cancer] again*,* but then I shouldn’t do*,* I’ve got to think of the positives. Leanne (D)*

Some appeared dubious about population screening and attending 3-yearly. Although trusting their updated risk and the clinician’s recommendations to attend for 3-yearly screening, women were apprehensive about the level of service they would encounter, as well as whether staff would be aware of their FHRPC involvement. For these women, 3-yearly screening would be a difficult transition despite understanding why they were no longer eligible. These concerns aligned with those who had previously been discharged from the clinic at age 60, who criticised population screening for being impersonal, with some not trusting negative mammogram results:

*I’m just going to be a number to them whereas I’m a name to [name of clinician] […] I walk into the one there and they don’t know my name*,* they don’t know me from Adam or Eve*,* they take longer to get the results back to you. It’s only every three years. Nobody ever discusses anything with you […] there’s no connection. Becky (D)*

In contrast, women in follow-up notified of a risk increase described experiencing a sense of relief due to remaining eligible for annual screening. Additionally, those who had been discharged and notified that their updated risk entitled them to receive annual screening were elated to be back in the FHRPC. For these women an increased risk was of course concerning, but this update seemed to act as an ‘entrance ticket’ or gateway back into the FHRPC. These women appeared to focus more on the positivity of additional surveillance, rather than the implications of the risk itself:

*It’s a double-edged sword*,* isn’t it? Your risk has gone higher*,* but guess what*,* you’re going to have it [mammography] once a year*,* yay. No*,* I’m actually quite happy about that*,* and I’m relieved*,* because if my risk level has gone up*,* obviously I then want as much preventative help*,* if you like*,* as possible*,* so I’m well happy to come once a year…Paula (I)*

Three women at the time of interviewing were taking preventative medication, with one woman, Leanne, advised to discontinue as a result of her updated risk. Leanne described feeling encouraged that taking preventative medication had appeared to reduce her risk and was positive about the prospect of being able to take a natural form of HRT to reduce her menopausal symptoms. Other women considered the thought of being asked to discontinue preventative medication hypothetically, with many explaining that although potentially worrying, medication was prescribed with the correct knowledge available at the time and not due to inaccurate information or medical negligence. Other women who had been offered preventative medication described it as *‘a no brainer’ (Hayley*,* I)* if it reduces risk but were cautious about side-effects, describing trusting the advice of the clinician but also needing to weigh up the ratio of harms to benefits before use.

## Discussion

Women who experienced a change in their clinically-derived breast cancer risk estimate reacted positively and trusted the estimate provided. A reduction in risk was met with relief as well as surprise, due to the previously perceived impact of family history on risk compared to other risk factors. Those who experienced a risk increase were not overly concerned by this, possibly due to seeing themselves as being at increased risk for most of their lives. Women described having a longstanding positive relationship with the FHRPC which appeared to influence the trust they had in their updated risk, as well as influencing the apparent ease at which they were able to integrate new risk information into pre-existing risk appraisals. A ‘gist’ understanding of new risk information seemed to be sufficient in enabling women to understand the ‘take home message’ of their breast cancer risk. Finally, for those at increased risk, updated estimates enable those under follow-up to remain so and facilitated a pathway back into the service for those previously discharged, resulting in positive reactions to this risk level. However, ineligibility for continued annual screening for those at reduced risk was difficult to reconcile.

### Relevance to existing literature

Women who were informed that their risk had increased accepted this news and did not appear overly concerned, nor did those referred back into the clinic blame the service for being discharged previously. Instead women here focused on their eligibility for annual screening and the elation of being referred back into the clinic. This contrasts with research suggesting that those given information about an increased risk or negative health outcome question the accuracy of the results and credibility of the source [23]. It is widely established that in the UK, cancer screening is highly valued [24]. Specifically, women living at chronic risk of breast and ovarian cancer view frequent screening as providing peace of mind and a sense of control [25]. Additionally, prior to diagnosis, those with breast cancer recalled having confidence in mammography, the clinic and their care [26]. In the present study the positivity of qualifying for more frequent screening outweighed the implications of a change in risk. This is in line with research that suggests although clinical risk consultations may contain messages of uncertainty and notifications of an increased risk, women tend to focus on the reassurance of the appointment and being a part of a ‘surveillance’ society [27].

Women’s appraisals of their breast cancer risk following their update were mostly in line with the clinical estimate provided. Women were able, with confidence, to attribute risk changes to their personal breast density, PRS or a combination, acknowledging the implications of these new risk factors. These findings are in contrast to those found with women from the general screening population who also received similar feedback where some women did not identify with their risk estimates, nor did they draw on specific risk factors to explain their risk [13]. However, there were significant differences in how risk was communicated in these two studies. For women in the general population, notification was received via letter, with a minority attending a consultation. Instead, established relationships with the clinic in the present study, as well as the *‘routine’* of discussing breast cancer risk may have influenced how open women were to receiving new risk information. This positive relationship with the FHRPC also seemed to positively impact women’s trust in the updated risk estimate provided, as well as influencing views on its credibility. It may also account for why those discharged felt apprehensive when faced with population screening, as relationships built at the FHRPC and comfort received would cease. This apprehension is also found in women offered risk estimation and stratification in the general population, with those previously receiving annual screening questioning the safety of 3-yearly screening and feeling less in control of their risk [28].

### Implications for practice

Women who were informed that their updated breast cancer risk estimate caused them to be ineligible for additional breast screening felt a sense of loss and were wary about population screening. These findings are consistent with literature that found that the recommendation to discontinue breast screening for older women (> 70 years of age) is met with resistance and scepticism, with some women feeling that this recommendation devalues their health [29, 30]. However, despite having some reservations, women who received a low-risk result from a breast cancer risk stratification feasibility study in the UK were more open to the idea of attending for screening less frequently, providing that the evidence is robust [31]. In the context of the present study, communication in FHRPCs needs to be carefully managed when women are discharged, with reasons thoroughly explained and concerns surrounding the perceived competence of population breast screening alleviated. For example, communication strategies designed to explain the discontinuation of screening for women aged 65 to 90 years showed that offering clear reasons for the change in recommendation, along with highlighting the benefits of this change, helped reduce cancer-related anxiety and decreased screening intentions [32]. From the present study it is also clear that FHRPCs should reinforce that staff (i.e. radiographers and radiologists) at population screening require the same level of training as those performing mammograms at FHRPCs. At population screening, radiographers should be made aware of women’s involvement in FHRPCs. Possessing this knowledge and demonstrating this to those recently discharged would perhaps reduce apprehensions and create a more personalised environment, making this transition easier.

It is understood that ‘gist’ health messages can increase patient satisfaction and are important for facilitating understanding and informed decision making around health risks [33, 34]. Understanding the ‘gist’ of their updated risk was sufficient for women to appreciate the most salient information from their consultations, resulting in mostly accurate risk appraisals and an appreciation of the impact of new risk factors. It has been recommended that risk communication research should move away from assessing patient’s verbatim representations of risk information and instead assess *‘gist’* representations, asking the question, *‘what does this risk information mean to the individual?*’ [34]. In clinical settings this level of understanding should be facilitated to enable patients to gain sufficient knowledge to inform ‘accurate’ personal appraisals of their risk and aid informed decision making. The use of visuals (e.g. breast density images) and analogies (e.g. like that given to explain SNPs/a PRS in the present study; see supplementary file) appear to be an effective way of facilitating this [35].

Women in the present study advocated for breast density notification for all. In the UK notification of personal breast density is only provided in research settings. Research from the US advises that education materials and breast density communication in clinical settings needs to be specifically addressed in order to help women make informed decisions about prevention management [36]. However, before communicating breast density information at a population level in the UK, it will be essential to reach consensus on the measures of breast density, as well as to clarify the harms and benefits of sharing this information with women and the prevention advice provided [37]. In the present study a PRS/SNPs information was attended to less than breast density, with a negative pathogenic variant test for high penetrance genes overshadowing the implications of a PRS. Healthcare professionals should consider providing written summaries of the implications of a PRS, or direct patients to a website to help facilitate knowledge acquisition once the affective response of receiving negative genetic test results subsides.

### Strengths and limitations

This is the first research study for any disease type to explore how a change in personal risk is understood, and reacted to in a clinical setting. It is important however to be cognisant that women interviewed were predominantly White-British, from one FHRPC and were seen by the same clinician employed at the clinic for the duration of women’s involvement with the service. Therefore, the relationship these women have with the clinic, which has been described as a ‘centre for excellence’, may not be representative of patient-clinician relationships in other FHRPCs or clinical settings worldwide, although it clearly shows that it is possible for patients to receive revised risk estimates without major adverse reactions. Furthermore, the women interviewed in this study were recruited from a self-selected sample of women within the broader FH-Risk study who chose to receive an updated breast cancer risk estimate. Therefore, it is important to note that women who opted not to receive an updated breast cancer risk estimate may have different perspectives on changes in their risk.

Findings in qualitative data analysis are at least partly a result of the subjectivity of the researchers generating and analysing the data [21]. The research team contributing to this work are from a range of disciplinary backgrounds, including health psychology, medical oncology and clinical genetics. The multi-disciplinary nature of this team resulted in meaningful discussions about the data, providing different perspectives on the themes generated. Prior to data generation, the research team had pre-existing apprehensions regarding reactions to a change in risk, especially if related to a change in preventative management. Although these concerns did not materialise, pre-conceived ideas were discussed openly prior to the interviews.

### Further research

Future research should establish how relationships between patients and doctors impact the effectiveness of risk communication and the trust patients have in updated risk estimates across different disease types and clinical settings. As well, research aiming to assess breast cancer risk appraisals across the entire FH-Risk cohort would be beneficial. This would facilitate an understanding as to whether risk appraisals are in line with the clinical estimates provided overall, as well as enable opportunities to explore how women view a changes in risk, as well as whether they have an understanding of the contributing risk factors. The longevity of ‘gist’ understandings and the impact this has on patient’s appraisals of a given disease risk and their informed decision making about prevention should also be assessed. Finally, future research should examine the clinical implications of communicating updated breast cancer risk estimates, including whether changes in women’s risk categorisation and subsequent management options lead to meaningful differences in health behaviours, clinical decision-making, and health outcomes.

## Conclusion

Women were positive about receiving a notification of change in breast cancer risk and trusted the estimate provided. This may have been due to a strong trust in the service communicating these updated risk estimates, facilitating an accurate appraisal of risk which was mostly in line with the clinical estimate provided.

## Supplementary Information

Supplementary Material 1.

Supplementary Material 2.

## Data Availability

The datasets generated during and/or analysed during the current study are not publicly available as they may contain information that would compromise participant consent, but are available from the corresponding author on reasonable request.

## Abbreviations

FHRisk: Family history risk study
FHRPC: Family history risk and prevention clinic
HRT: Hormone replacement therapy
NICE: National institute for health and care excellence
PPIE: Patient and public involvement and engagement
PRS: Polygenic risk score
SNPs: Single nucleotide polymorphisms
