# Supplementary material for: How do women experience a change in their clinically-derived breast cancer risk estimates: views from a UK family history risk and prevention clinic
Source: BMC Cancer. 2026 Jan 29;26:287. doi: 10.1186/s12885-026-15651-w (PMC12924285; doi:10.1186/s12885-026-15651-w)
Supplement: Supplementary file 1 — Supplementary Material 1. [file 12885_2026_15651_MOESM1_ESM.docx]

**Updated risk consultation summary letter**

Dear

**Re: Exploring the experience of receiving updated breast cancer risk estimates in women with a family history of breast cancer: communicating a revised risk estimate**

Thank you for taking the time to attend your risk consultation appointment about the reassessment of your breast cancer risk. The aim of the conversation was to let you know how your risk may have changed and whether this alters our advice about screening and prevention. This letter summarises what we spoke about.

Your given risk when you first attended the clinic was a 40% chance of developing breast cancer. This risk was estimated from the standard models we used at that time and using your family history, hormonal and lifestyle factors.

As we discussed, your risk may change with the passage of time but may also become more accurate, when we include factors like your breast density (the ‘whiteness’ of the mammogram) and DNA tests (SNP score and gene test).

**Your breast density** on your latest mammogram was recorded as “C”, moderate density and is pointed out on the diagram below. This degree of density increases your risk.

**Your breast density**

**
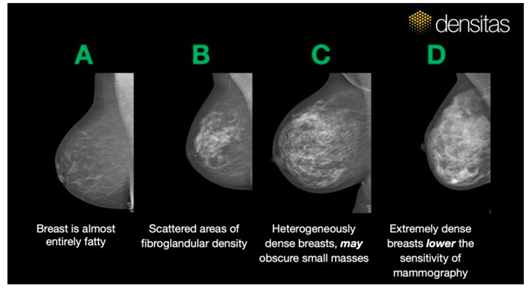
**

**Your SNP score** is 2.1 (average 1.0) which increases your risk.

**Your test** for 12 genes related to breast cancer and 21 to other cancers is negative.

**Overall**

We put all this information above together to get an indication of the change in your risk since your first visit to the clinic. Overall, your risk has increased.

In the next 10 years your risk of developing breast cancer is 16.8% compared with 3.1% in the general population.

During your lifetime your risk is 42% compared with 10.8% in the general population. Thus, you have about four times the population risk.

**What are your options to reduce your risk?**

The following things can be done to reduce your risk of developing breast cancer:

Screening – [insert here]

Lifestyle change – [insert here].

Medication for preventions – [insert here]

**In summary**

Since your first visit to the clinic your risk has increased because of a high SNP score and moderately high breast density.

We are very grateful for your help with the FH-Risk studies. In turn we hope that you feel they have helped you.

With best regards,

**Example of PRS/SNP analogy women received**

*We have found over 300 places along your DNA which are related to your risk of breast cancer (These are called single nucleotide polymorphisms or SNPs (pronounced SNIPS) for short. We have measured all of these sites in your blood sample. At some of the 300 places there is a change which results in a small increase risk of breast cancer which we call A for the sake of illustration. Changes at other places may result in a small decrease in risk which we call B. If you have more As than Bs you are at overall increased risk. If you have more Bs than As you are at decreased risk. Most women have about the same number of As and Bs so there is little change in risk. Others may have more of one than the other, which can result in markedly higher or lower risk. We add these results to your standard risk factors and your breast density to come up with an overall risk score as will be outlined in our letter to you. Putting SNPs together with all the other risk factors helps us give a more precise risk score for you which helps decide your continued need for screening and whether to suggest preventive therapy. So with all this in mind, we have found that you have more As than Bs, meaning that your risk has slightly increased on this point.*

**Interview questions and topic areas (to be used flexibly)**

***General questions about risk from the first FH-Risk study***

1. In as much detail as you can, can you tell me what you remember about receiving your risk of breast cancer from the FH-Risk study you took part in around 10 years ago?
   1. Can you tell me what you remember about the risk information you were given?
   2. Do you remember how you felt?
   3. How have your views on your risk changed since you received that initial risk information? Why/why not?
   4. Did you change anything about your health behaviours in response to this initial risk information? Why/why not?

***Prior to the consultation:***

1. I’d like to move on now and talk to you about your most recent consultation where you received an update about your risk. So I’d first like to start by asking you about the letter inviting you to the family history clinic to receive your updated breast cancer risk. How did you feel when you received this letter?
   1. Why did you feel that way?
   2. Did you have any concerns before your appointment? Why/why not?
   3. How did you feel about the possibly of receiving new risk information?
   4. Did you ever think that breast cancer risk could change? What did you think?
   5. Did you think that your risk of breast cancer may have changed? Why?
   6. How were you feeling before the appointment? Why were you feeling this way?

***The risk consultation:***

1. Could you tell me in as much detail as you can about the appointment you had a few weeks ago where you discussed your breast cancer risk?
   1. Can you remember what he/she told you?
   2. What did you think about the risk you were given?
   3. How did receiving your risk make you feel?
2. How was your risk explained to you?
   1. What did you learn about your risk?
   2. Is there anything that you are still unsure about?
   3. Did you have any concerns about the information provided? Why/why not?
3. How do you personally feel about your risk now?
   1. Can you tell me how you personally think about your breast cancer risk?
   2. Having had time to reflect on the information you were given in the risk consultation, have your views on your breast cancer risk changed? Why/why not?

***Understanding changes to risk:***

1. What do you understand about why your risk might have changed [or stayed the same]?
   1. What factors do you think caused your risk to change [or stay the same]?
   2. Do you have any specific concerns about your breast cancer risk, e.g. understanding exactly why the estimate has gone up/down/stayed the same?
2. Are you aware of what breast density is?
   1. How best would you describe breast density?
   2. Do you believe the term breast density was explained to you in your risk appointment?
      1. [if no – provide definition]
      2. [if yes ask – what do you think about how this was explained by the doctor?]

[Breast density definition if needed: *When we look at breast density from a mammogram we are looking for the amount of tissue that isn’t fat in your breast. The more tissue you have in your breasts that isn’t fat the denser your breasts are. Denser breasts equals a higher risk of breast cancer. Also this tissue in the breast appears white on mammograms and so does cancerous masses. This can mean that dense breast tissue can obscure cancer, making it difficult for us to spot. Does that make sense? Do you have any questions about that? What do you think about this (if anything)?]*

- 1. Do you have any concerns about breast density?
  2. [If applicable] do you think your risk changed due to your breast density? Why/why not? How does knowing that make you feel?

1. Do you remember if the doctor discussed genetic information with you? What can you recall anything about this?
   1. Do you have any concerns about the genetic information provided?
2. What information could we give women who attend the clinic to help them understand their risk, especially if it has changed?
   1. In what formats/ways could we do this? (prompt: leaflets, letters, hotline service etc.)

***Risk reduction:***

1. Do you think receiving your updated risk will affect the decisions you make to reduce your risk? Why/why not?
2. Are you aware that some women at above average and high risk can take medication to reduce their risk?
   1. [If yes] where have you heard about risk reducing medication?
   2. Has it ever been offered to you? And what did you think?
   3. Why did/didn’t you decide to take the medication?
   4. [If applicable] In light of what you were told at your most recent risk consultation would you consider taking medication to reduce your risk? Why/why not?
3. Some women who have received an updated breast cancer risk estimate may not be eligible for extra screening anymore. How would you feel if you were no longer eligible for extra screening? [change question if this situation does apply to a woman]
4. Some women may have also been taking medication to reduce their risk when they didn’t need to. How would you have felt if this had been your situation? [change question if this situation does apply to a woman]

***Overall thoughts:***

1. Do you think your most recent breast cancer risk estimate reflects how you personally feel about your breast cancer risk? Why/why not?
2. Do you have any concerns about your updated breast cancer risk estimate?
   1. Do you trust the estimate provided?

**Finishing comments**

Thank you for your time today. We really appreciate it.

- Is there anything you would like to add?
- Is there anything you think we would have covered and we haven’t?
- Is there one important thing that you would like us to take from this?
- Do you have any questions for me?
